# Serum Biomarkers of Extracellular Matrix Remodeling in Ulcerative Colitis—One Step Closer to Fibrosis Biomarkers in Inflammatory Bowel Disease

**DOI:** 10.1002/ueg2.12770

**Published:** 2025-02-13

**Authors:** Helena Tavares de Sousa, Raquel Oliveira

**Affiliations:** ^1^ Gastroenterology Department Unidade Local de Saúde Algarve Portimão Portugal; ^2^ Faculty of Medicine and Biomedical Sciences University of Algarve Faro Portugal; ^3^ ABC—Algarve Biomedical Center University of Algarve Faro Portugal; ^4^ School of Medicine Life and Health Sciences Research Institute (ICVS) University of Minho Braga Portugal

Fibrosis is a serious complication of both Crohn's disease (CD) and ulcerative colitis (UC) and is currently the major unmet need in inflammatory bowel disease (IBD) management [1]. Although much less prevalent in UC, strong evidence supports severity and chronicity of inflammation are the main drivers of excessive extracellular matrix (ECM) deposition in the submucosa and muscularis mucosae [2], leading to increased wall stiffness, and ultimately to motility and anorectal dysfunction, rectal urgency and incontinence [3].

In recent years knowledge about inflammation‐dependent and ‐independent fibrogenesis in IBD has increased dramatically due to intensive worldwide multiomics research on inflammation and fibrosis, mostly in CD but also in UC and animal models of colitis. Fibrosis, defined as excessive and uncontrolled deposition of ECM, results from a complex process involving immune and non‐immune cells, their soluble mediators, and exposure to luminal contents, such as microbiota and environmental factors [1]. High‐resolution techniques like single‐cell RNA sequencing (scRNA seq) or spatial transcriptomics, that systematically map and characterize tissue cell sets and subsets according to their frequencies, gene expressions, cell‐cell interactions and so on, have uncovered the cellular compartment [4, 5, 6, 7, 8]. Particularly, several colonic fibroblast subsets have been recognized. A specific subset termed inflammation‐associated fibroblasts (IAFs) was identified in IBD inflamed tissue, expressing genes associated with inflammation, fibrosis and even cancer [6, 8]. Interestingly, persistence of IAFs correlated with failure of anti‐TNF and vedolizumab therapy [4, 6], while their decrease and reduced fibrosis gene expression were observed in responders to vedolizumab [4] and ustekinumab [9].

The study by Venkat and colleagues [9] aimed to identify novel serum biomarkers associated with intestinal IAFs (IIAFs), tissue remodeling and treatment response to ustekinumab in UC. The authors propose that such peripheral biomarkers reflective of tissue stromal subsets would be useful in developing stromal modulators. Moreover, these biomarkers would also potentially work as an early non‐invasive identification of fibrosis, which is also a major gap in the clinical monitoring of IBD patients [1].

To this aim, Venkat and colleagues used colonic tissue retrieved from formalin‐fixed paraffin‐embedded (FFPE) colonic biopsies from 91 UC participants in the UNIFI trial and serum and matched biopsies data from weeks 0 and 8 to correlate the biomarkers with the IIAFs tissue biology.

Using, among others, bulk tissue and scRNA seq, authors identified an IIAF's subset that significantly associated with inflammatory activity as measured by endoscopic Mayo and Geboes scores. Importantly, IIAFs were significantly reduced in clinical responders after ustekinumab treatment at week 8. No data was presented regarding the correlation of IIFAs with inflammatory biomarkers, such as fecal calprotectin. Interestingly, IIAFs exclusively expressed several matrix remodeling genes. Hence, beyond inflammation, IIFAs were also associated with transcriptional profiles reflective of tissue remodeling.

As for the serum biomarker part of the study, a Protein Fingerprint assay assisted in the identification of protein fragments of collagens (C1M, Pro‐Collagen 22, CTX‐II) and elastin (EP‐3) in the same matched UNIFI serum samples. A significant correlation was noted between Pro‐Collagen 22, C1M and ELP‐3 and IIAFs; and between Pro‐Collagen 22, C1M and CT‐III and levels of collagens and MMPs. However, although Pro‐Collagen 22 and C1M significantly decreased after 8 weeks of ustekinumab treatment, none of the four biomarkers correlated with UC inflammatory activity as measured by fecal calprotectin. Hence, authors proposed this set of serum biomarkers to be distinct from other existing biomarkers, reflecting a defined cellular subset biology in UC, namely the IIFAs. This would explain the biomarkers' decrease after ustekinumab treatment. Venkat and colleagues concluded having identified peripheral protein biomarkers that could provide a non‐invasive approach to evaluate stromal cellular tissue biology and predict patients' risk for IIAF‐associated fibrotic disease.

This study raises some interesting issues that should be addressed in future studies.

It is an undoubtedly valuable investigation, identifying a specific subset of fibroblasts related to inflammatory activity and matrix remodeling serum biomarkers, namely products of collagen and elastin cleavage in UC. However, these are not necessarily markers of fibrosis, as ECM turnover is not a synonym of fibrosis. The stromal compartment of the intestinal wall consists mainly of ECM and stromal cells, which physiologically restore the damaged epithelium while maintaining a continuous matrix remodeling that assures its homeostasis. Fibrosis emerges when excessive and uncontrolled production and deposition of ECM occurs [1, 10]. Hence, further studies are needed to investigate the correlation between IIFAs and these serum biomarkers of matrix turnover with colonic fibrosis in both UC and CD. Additionally, the absence of correlation studies between IIFAs and proven inflammation biomarkers, after demonstrating their significant association with endoscopic and histologic activity, demands attention in future studies.

Overall and most importantly, the study by Venkat and colleagues presents a significant advancement in noninvasive biomarkers of matrix remodeling in UC, which have the potential to become part of the much‐needed noninvasive biomarkers of intestinal fibrosis in IBD.

## Conflicts of Interest

The authors declare no conflicts of interest.

## Data Availability

The data mentioned in this text can be found in the corresponding reference.
